# P-456. Acute Mastoiditis in Children in the era of Pneumococcal Conjugate Vaccines

**DOI:** 10.1093/ofid/ofaf695.671

**Published:** 2026-01-11

**Authors:** Nahed M Abdel-Haq, Ronald Thomas, Basim Asmar

**Affiliations:** Children's Hospital of Michigan. Central Michigan University, Detroit, MI; Central Michigan University, Detroit, Michigan; Children's Hospital of Michigan. Central Michigan University, Detroit, MI

## Abstract

**Background:**

Acute mastoiditis (AM) is a complication of otitis media. The most common cause is *Streptococcus pneumoniae*. The incidence of invasive pneumococcal infections has decreased following the introduction of PCV-7 in 2000 and PCV-13 in 2010. We evaluated the changes in the incidence, bacteriology and complications of AM following introduction of PCV-7 and PCV-13.

**Methods:**

A retrospective review of medical records during 19-year period (2000-2018). AM was defined as a suppurative infection of mastoid air cells with symptoms of less than 3 weeks.

**Results:**

We identified 242 children with AM: 138 in the first period (2000 to 2010): PCV-7 and 104 in the second period (2011 to 2018): PCV-13. No significant change in AM incidence was noted: 11.0 cases/10,000 admissions in the first period and 8 cases/10,000 admissions in the second (p=0.31). Ages ranged from 3 months to 18 years (median 5.6 years). Symptoms included fever (52%), ear pain (63.6%) and mastoid tenderness (64.9%). Prior antibiotics were prescribed to 37.6%. Surgery was performed on 52.1%. Cultures from 125 patients showed *S. pneumoniae* (29.6%), *S. pyogenes* (17.6%), *P. aeruginosa* (16.8%), *S. aureus* (13.6%). Two had complicated AM due to *Fusobacterium necrophorum*. During the second period, *S. pneumoniae* was detected on 14/46 (34.8%) compared to 21/79 (21.1%) in the first (p=0.417). Partial or complete PCV vaccination was documented in 89 patients including 13/37 who had S. pneumoniae culture-positive AM. Most common parenteral antibiotics used were: IV ceftriaxone (64.5%), IV clindamycin (42.1%), IV ampicillin/sulbactam (19.8%). Most common oral antibiotics: amoxicillin-clavulanate (38%), clindamycin (20.2%) and amoxicillin (8.3%). Complications included bony erosive changes (mastoid/temporal bone) in 78, soft tissue abscesses in 39, venous sinus thrombosis in 10, facial nerve palsy in 6, intracranial extension in 7 patients, 6 of which in the second period.

**Conclusion:**

Despite a decrease of invasive pneumococcal infections post PCV-7, the incidence of AM in our pediatric population remained unchanged after PCV-13 introduction with many complications. The role of the updated pneumococcal conjugate vaccines on AM incidence needs further evaluation.

**Disclosures:**

All Authors: No reported disclosures

